# Supplementary material for: Robust estimates of overall immune-repertoire diversity from high-throughput measurements on samples
Source: Nat Commun. 2016 Jun 15;7:11881. doi: 10.1038/ncomms11881 (PMC4912625; doi:10.1038/ncomms11881)
Supplement: Supplementary Information — Supplementary Figures 1-4, Supplementary Methods and Supplementary Reference [file ncomms11881-s1.pdf]

Supplementary Figures

Supplementary Figure 1

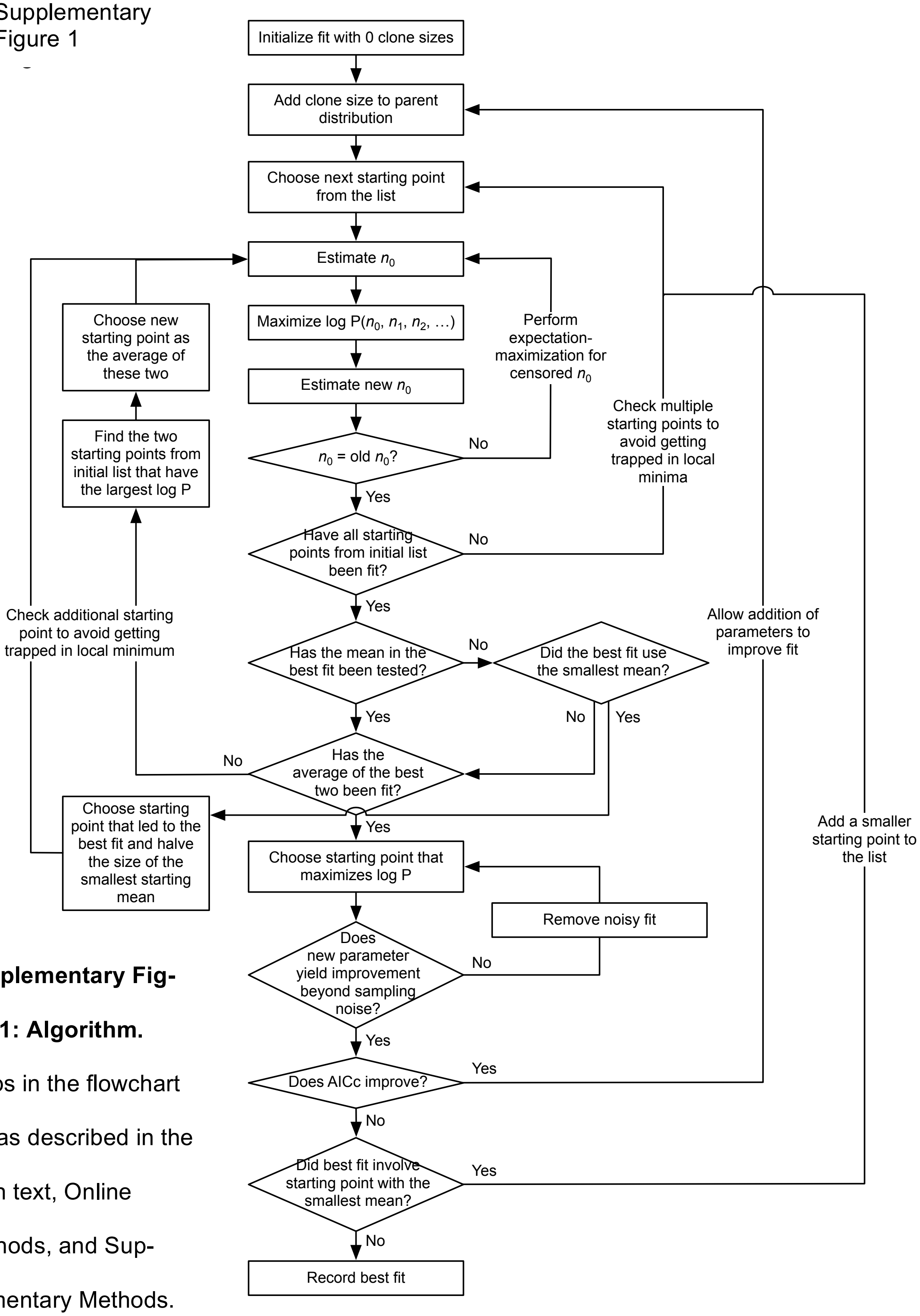

Supplementary Figure 1: Algorithm. Steps in the flowchart are as described in the main text, Online Methods, and Supplementary Methods.

Supplementary Figure 2a-c

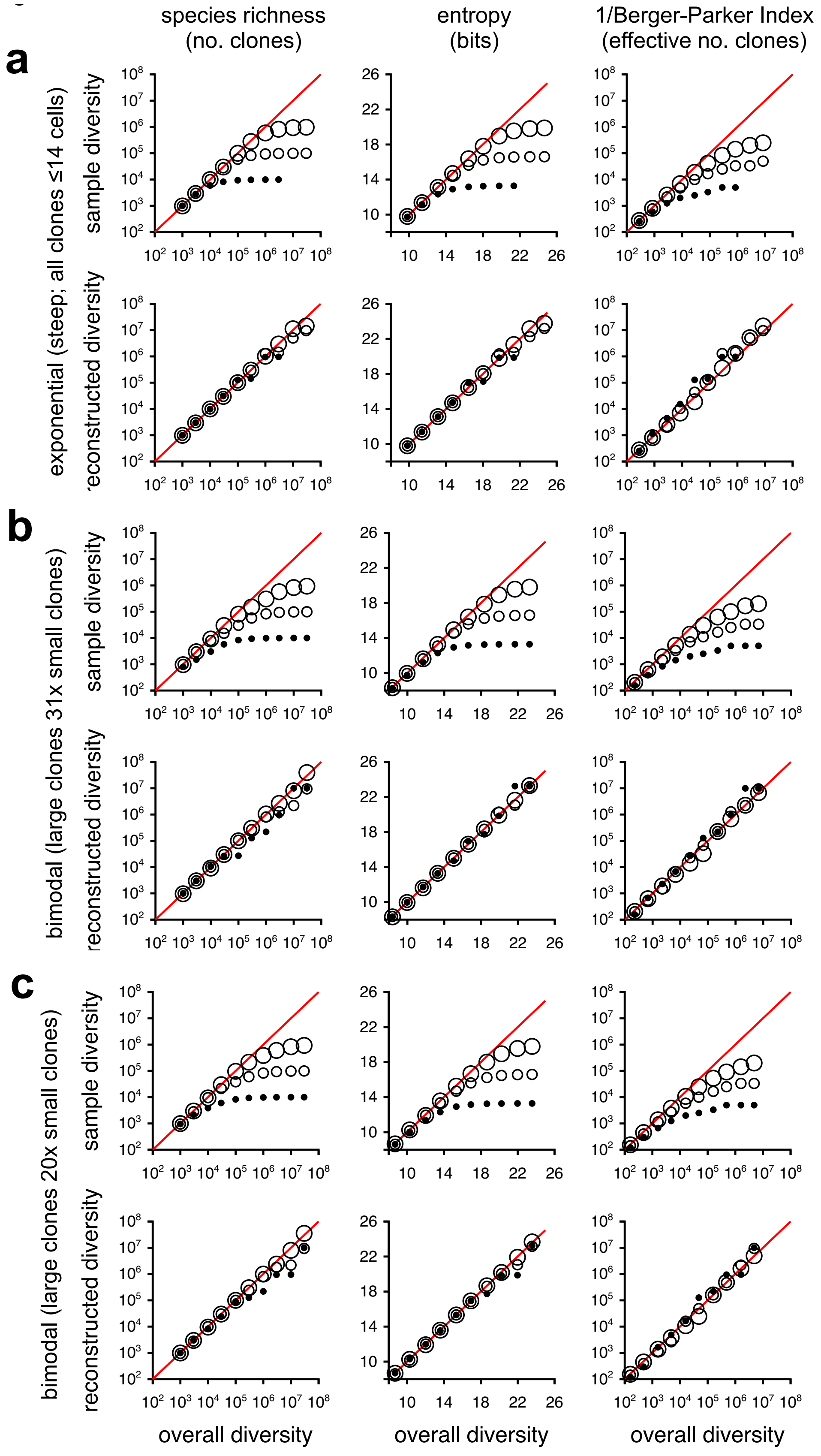

Supplementary Figure 2d-g

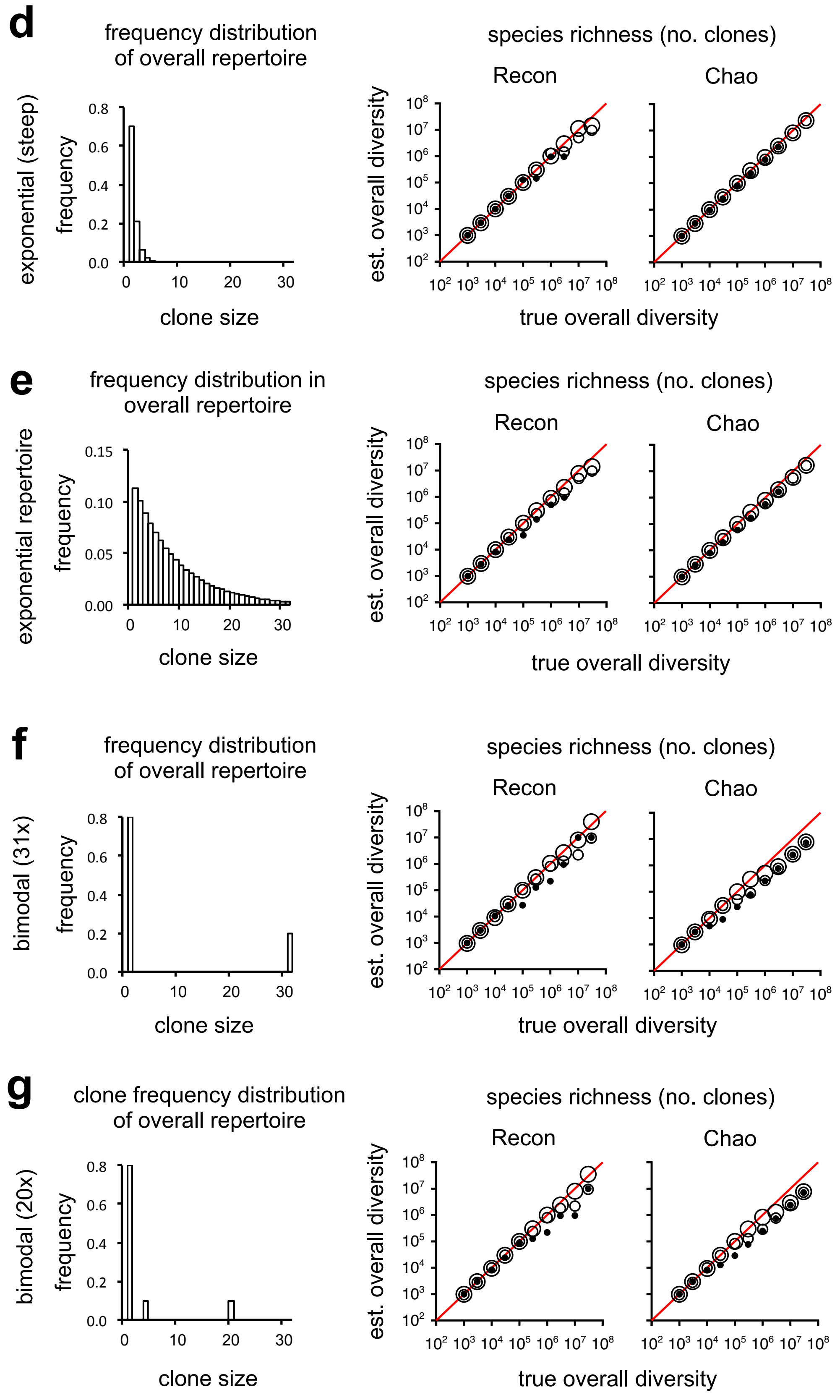

Supplementary Figure 2: Comparison to other algorithms. Recon diversity vs. other estimates showing fits to additional gold standard repertoires plotted as for Figure 2. (a)-(c) Comparisons of sample diversity (top) to Recon diversity (bottom) plotted as in Figure 2a for (a) a steep exponential clone size distribution (b) a bimodal distribution in which the overall distribution contains a population of small clones and a population 31 times as large and (c) a bimodal distribution in which the overall distribution contains a population of small clones and a population 20 times as large. (d)-(g) Comparison of species richness estimates by Recon (middle) and CE (right) shown as in Figure 2b for an example additional gold standard overall distributions (left) for (d) a steep exponential clone-size distribution, (e) a shallow exponential clone-size distribution, (f) a bimodal distribution in which the overall distribution contains a population of small clones and a population 31 times as large, and (g) a bimodal distribution in which the overall distribution contains a population of small clones and a population 20 times as large.

Supplementary Figure 3

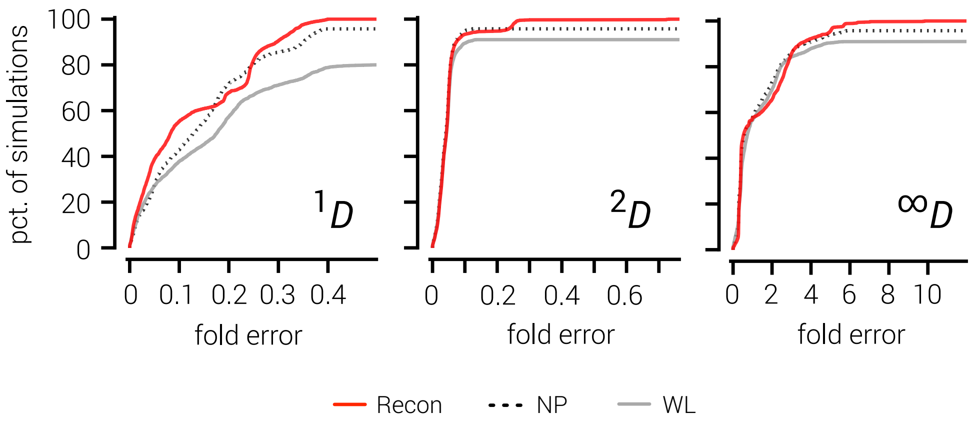

Supplementary Figure 3: Recon vs. CE, NP, and WL on noisy distributions. Each pair of cumulative distributions show accuracy (left) and speed (right) for 100 realizations of noise on the different distribution types described in Fig. 2.

Supplementary Figure 4

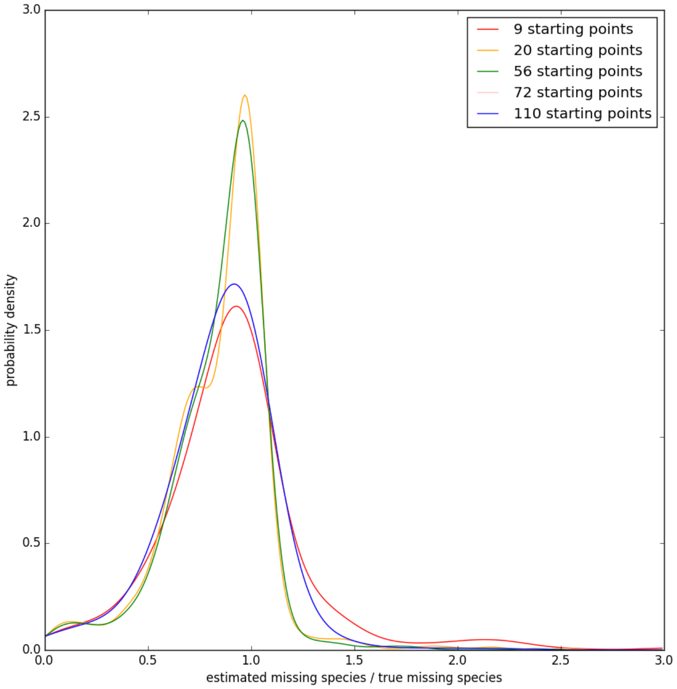

Supplementary Figure 4: Scanning. Probability densities of the ratio of estimated missing species/true missing species demonstrating the benefit of using additional starting points. Fits using, in each round of fitting, 9 (red), 20 (yellow), 56 (green), 72 (pink) and 110 (blue) combinations of starting weights and means (yellow) show that the set of 56 starting points used in the main study result in a sharper peak of the probability distribution function (pdf) near 1.0, and diminished trapping in local minima away from 1.0. Pdfs are plotted using Gaussian kernel density estimates over 800 samples from gold-standard distributions (see main text).

Supplementary Methods

Detailed description of the Recon algorithm

Overview. The problem is, given the observation of the clone size distribution in a sample, to reconstruct the number of clones of each size in the parent or overall population from which a sample was taken (e.g. memory B cells in the peripheral blood).

By *clone size* we mean the number of cells that make up a clone. A clone made up of a single cell has clone size 1, while a clone made up of a million cells has clone size one million.

By the *clone size distribution* we mean the number of clones of each size (Fig. 1a). For the sample we use the notation n_i, where i indexes the clone size and n_i is the number of clones of that size. Thus n_1 is the number of clones represented in the sample by a single cell, n_2 the number of clones represented by 2 cells, and so forth. The number of clones that are present in the parent distribution but missing from the sample is represented by n_0, as they are represent-ed by 0 cells in the sample. These are the *missing species*.

Experimentally observed clone size distributions are described by a sampling distribution from the parent population. The overall strategy of the Recon algorithm is to find a maximum-likelihood estimate (MLE) for parameters of a model describing the sampling distribution. The form of the model has an immediate interpretation in terms of the clone size distribution of the parent population.

Recon is based on a mixed-Poisson model for the contribution of each clone in the parent population to the sample:

$$p_i = \sum_j w_j \, Poisson(i;\, m_j)$$

$$= \sum_j w_j \frac{m^i \exp(-m_j)}{i!}$$

where w_j are *weights* and m_j are Poisson parameters. The weights w_j give the proportion of clones in the parent population with clone size j.

The parameters m_j give the mean number of cells a clone of size j contributes to a sample and are referred to as *means* below. They correspond to clone sizes in the parent population:

$$\text{Clone size } j \text{ in parent} = \text{number of cells in parent population} \times m_j / \text{ sample size}$$

The parameters therefore give a complete description of the clone-size distribution in the parent population.

If there are k different sizes in the parent population, so that the index j ranges from 1 to k, then there are a total of $2k$-1 independent parameters, consisting of k independent sizes m_j and k-1 independent weights w_j, which sum to 1.

Assuming that the sample comes from a well mixed parent population, such as blood, this gives rise to a sampling distribution:

$$P(n_0, n_1, n_2 \ldots) = (n_{\text{total}}!/n_0!\, n_1!\, n_2! \ldots) \times (p_0^{n_0} p_1^{n_1} p_2^{n_2} \ldots) \qquad (1)$$

Where n_{total} is the sum of all n_i.

The Recon algorithm addresses three fundamental problems in the search for parameters which maximize the likelihood (1) given the data n_i.

First is the need to determine the number of different clone sizes, k. This is addressed by starting with a homogeneous population in which all clones are the same size and refining the description of the population by adding clone sizes (incrementing k by 1) until no better fit can be obtained. A better fit must both (*i*) improve the fit by an amount that is larger than expected variation from sampling noise and (*ii*) improve the corrected Akaike Information Criterion (AICc). This loop is described in Steps 2, 7 and 8 below.

Second is that the likelihood is a non-linear function of the parameters and has local minima, whereas a global minimum is desired. In practice, for some fits, the AICc allows 20 or more parameters; searching such a high dimensional space requires a careful strategy to find a global minimum. To handle this problem, in Recon each step of the fit is run many times (nine in our implementation) from different starting points. These multiple different starting points often result in finding multiple local minima, from which the global minimum is selected. This loop is described in Steps 3 and 6 below.

Third, the likelihood of the data can be calculated directly from the $2k$-1 parameters of the mixed-Poisson distribution model given only the number of unseen species, n_0. Thus, the number of missing species must be jointly modeled as an additional parameter.

In order to handle this problem an expectation maximization (EM) approach (see references in main text) is used in which an expected value of n_0 is obtained from the remaining parameters and parameters are then refitted until self consistent values of parameters and of n_0 are obtained. This loop is described in Steps 4 and 5 below.

These three nested loops are shown in the flowchart in Supplementary Figure 1.

Step 1: Separate large from small clones. To simplify our calculations of n_0, the first step splits the observed clone size distribution into large clones and small clones. Our implementation uses a threshold of 30 cells.

Consider repeated sampling of the parent distribution. Any clone in the parent population that is large enough to contribute 30 cells to a sample will essentially always be represented in the sample; i.e., it will never contribute to the number of missing clones, n_0. Furthermore, the sampling error on such large clones will be relatively small, and the size of the clone in the parent population will scale linearly with the number of clones in the sample.

The main work of reconstruction must then be applied to the remaining small clones, whose contribution to the observed sample is less than 30 cells, and which correspond to clones in the parent population that are small enough to include clones that will contribute no cells to the sample and thus affect n_0. The remaining reconstruction steps are applied only to these small clones.

Step 2: Determine mean observed clone size. The mean size of all observed clones contributing to the fit (i.e. clones contributing less than 30 cells) is calculated. This is used to set the scale for initial guesses of clone sizes in step 3.

The initial parameters for the fit are set to empty lists of weights and means. This is recorded as the *current best fit*.

Step 3: Add a clone size to the parent distribution. Next, the algorithm adds a new distinct clone size to the parent population, in such a way that the new distribution maximizes the log likelihood. Because there are multiple maxima in the likelihood, this fitting (Steps 3-5) will be repeated for each of many starting points for the new clone size added to the same current best

fit in an attempt to find the best possible improvement. We used nine starting points in our implementation.

Except on the first iteration of the fit, the weight of the new clone size is selected from the list of starting weights (Supplementary Table 1). On the first iteration of the fit the newly added clone size is the only clone size, so the weight is 1.0. The mean for the new population is calculated by selecting a starting scale factor (Supplementary Table 2) and multiplying by the mean size of the small clones.

Supplementary Table 1: Starting weights
0.05
0.128
0.207
0.286
0.364
0.443
0.521
0.6

Supplementary Table 2: Starting scale factors
0.05
0.225
0.4
0.575
0.75
0.925
1.1

The number of missing species is updated as

$$n_0 = n_{obs}/(1 - p_0)$$

where n_{obs} is the number of small clones observed in the sample (i.e. the sum of n_i for $0<i<30$).

Step 4: First EM step. Given the estimate of n_0, Recon maximizes log P, where P is given by Eq. (1) above:

$$P(n_0, n_1, n_2 \ldots) = (n_{\text{total}}!/n_0!\, n_1!\, n_2! \ldots) \times (p_0^{n_0} p_1^{n_1} p_2^{n_2} \ldots)$$

The n_i for $i>0$ are the observed number of clones represented by i cells in the sample, n_{total} is the sum of all n_i including n_0, and the p_i are the probabilities of a randomly selected clone giving rise

to exactly n_i cells in the sample, as calculated from the mixed-Poisson model. In our implementation this is carried out using the L-BFGS-B minimization method from the scipy.optimize library.

Step 5: Second EM step. A new value for n_0 is estimated according to:

$$n_0 = n_{obs}/(1 - p_0)$$

.

This new value of n_0 is used to find maximum likelihood values for the parameters.

If the newly estimated value of n_0 is equal to the old value of n_0 then there has been no improvement, and so EM for the corresponding starting point is completed.

If instead the newly estimated value of n_0 differs from the old value of n_0 then Step 4 is repeated using the new estimate and starting from the parameter values given by the fit for the old n_0 estimate. This ensures that the end result of EM is a set of parameters that maximize likelihood and produce a self-consistent estimate for n_0.

The result is added to a list of *possible best fits.* As shown in Supplementary Figure 1, the algorithm returns to Step 4 until all starting points have been tried and the list of possible best fits contains nine entries. Note that at this point the current best fit is not yet updated.

6: Compare the multiple minima that arise from the different starting points. After all nine fits, each starting from different initial parameters, are complete, the MLE from among these nine fits is selected.

The likelihood minimized in Step 4 treats n_0 as data, and maximizes likelihood given that data. However, solutions from different starting points will arrive at differing self-consistent values of n_0. In order to compare these solutions n_0 must be treated as a parameter rather than as data.

Treating n_0 as data we use Eq. (1) above. For practical purposes, since the n do not depend on the parameters, we maximize

$$\log(p_0^{n_0} p_1^{n_1} p_2^{n_2} \ldots) = \sum_{i=0}^{\infty} n_i \log p_i$$

Because the p_i are known functions of the mixed-Poisson model parameters this is a straight-forward procedure.

In contrast, treating n_0 as a parameter we have the likelihood to be maximized:

$$P'(n_1, n_2, n_3, \ldots | n_0) = \left(\frac{n_{\text{obs}}!}{n_1!\, n_2!\, n_3! \ldots}\right) \times (p'^{n_1}_1 p'^{n_2}_2 p'^{n_3}_3 \ldots)$$

Here the p'_i are not equal to p_i, (as can be seen e.g. by considering normalization) and depend on n_0. It is not straightforward to calculate the p'_i from the mixed-Poisson model parameters.

In order to calculate P' in terms of the mixed-Poisson model parameters we write log P' in terms of log P:

$$P(n_0, n_1, n_2 \ldots) = \left(\frac{n_{\text{total}}!}{n_0!\, n_{\text{obs}}!}\right) p_0^{n_0} (1 - p_0)^{n_{\text{obs}}} \left(\frac{n_{\text{obs}}!}{n_1!\, n_2!\, n_3! \ldots}\right) \times (p'^{n_1}_1 p'^{n_2}_2 p'^{n_3}_3 \ldots)$$

$$= \left(\frac{n_{\text{total}}!}{n_0!\, n_{\text{obs}}!}\right) p_0^{n_0} (1 - p_0)^{n_{\text{obs}}} P'$$

Then

$$\log P = \log\left(\frac{n_{\text{total}}!}{n_0!\, n_{\text{obs}}!}\right) + n_0 \log p_0 + n_{\text{obs}} \log(1 - p_0) + \log P'$$

so

$$\log P' = \log P - \log\left(\frac{n_{\text{total}}!}{n_0! n_{\text{obs}}!}\right) - n_0 \log p_0 - n_{\text{obs}} \log(1 - p_0). \qquad (2)$$

Taking the log of Eq. (1) we can write

$$\log P = \log\left(\frac{n_{\text{total}}!}{n_0!\,n_1!\,n_2!\,\ldots}\right) + n_0 \log p_0 + \log\left(p_1^{n_1} p_2^{n_2} p_3^{n_3} \ldots\right).$$

Substituting this expression for log *P* into Eq. (2) we find:

$$\log P' = \log\left(\frac{n_{\text{obs}}!}{n_1!\,n_2!\,n_3!\,\ldots}\right) - n_{\text{obs}} \log(1 - p_0) + \log(p_1^{n_1} p_2^{n_2} p_3^{n_3} \ldots)$$

The first term on the right does not depend on parameters, so in order to maximize *P'* we select the fit giving the maximum value of:

$$\log(p_1^{n_1} p_2^{n_2} p_3^{n_3} \ldots) - n_{\text{obs}} \log(1 - p_0).$$

Because this is written in terms of the p_i it can be evaluated in terms of the mixed-Poisson model parameters, so it is straightforward to maximize. Note that directly comparing the log likelihoods treating n_0 as data between fits that have different values of n_0 is in practice misleading, and leads to a severe bias against large values of n_0.

The 2 fits with the highest log likelihoods are passed to Step 7

Step 7: Fit using smaller initial mean

If the best fit from Step 6 (i.e. the fit with the highest log likelihood) started from the smallest initial mean, then the starting weights and means that lead to the best fit are taken and the smallest mean is halved in value. These starting weights and means are then fit using Steps 4 and 5 of the algorithm.

The log likelihood of the resulting fit is computed as in Step 6.

If the best fit passed from Step 6 did not start from the smallest initial mean, then proceed directly to Step 8.

Step 8: Fit average of best starting points

The starting weights that led to the two best fits are then averaged together to produce the best average starting weights. The starting means that led to the two best fits are averaged together to produce the best average starting means. The best average starting weights and means are then fit using Steps 4 and 5 of the algorithm.

The log likelihood of the resulting fit is computed as in Step 6.

The resulting 57 or 58 fits ordered from highest to lowest log likelihood is passed to Step 8 as the list of *candidate best fits*.

Step 9: Check sampling noise and minimum clone size. If an estimate of the number of cells in the parent population is available then it is possible to set a minimum size for clones in the parent population—namely 1 cell. However, in general such estimates may not be available, and the Recon algorithm does not rely on such information.

If there is no restriction on the minimum clone size then the algorithm can produce a perfect fit to n_1 in the observed clones by fitting a large number of clones, each of which contributes an unrealistically small fraction of a clone to the observed distribution. It is therefore necessary to introduce a minimum mean clone size.

The expected number of cells contributed to n_1 by the clones with the smallest m parameter in the candidate best fit is compared against the expected noise in n_1 arising from the remaining clones. In our implementation, the noise threshold on the remaining clones is calculated as three times the standard deviation from Poisson sampling.

If the contribution from the smallest clones in the candidate best fit with the highest log likelihood is larger than this noise threshold then it is passed to Step 10.

Otherwise, it is removed from the list of candidate best fits and the next candidate best fit is tested until a fit is found for which the contribution from the smallest clones is larger than the noise threshold. This fit is then passed to Step 10.

Step 10: Test for improvement of the AICc. The AICc is defined as

$$\mathrm{AICc} = 2q - 2\ln P' + 2q(q+1)/(N-q-1)$$

Where q = 2k-1 is the number of parameters and N is the number of observations. N is taken as the number of distinct clone sizes that being fitted (plus the two nearest observations of zero clones, if applicable), which in the case of Recon is limited by the number of small clone sizes, i.e. ≤29 in our implementation.

The new AICc of the new candidate best fit is compared against the AICc of the current best fit. Note that in this step the candidate best fit has two more parameters (one weight and one population size) than the current best fit. This is what necessitates the use of the AICc. (In previous steps, comparisons were made only between fits with the sample number of parameters, so a simple log likelihood comparison sufficed.)

If the candidate best fit is not an improvement then the algorithm exits with the current best fit as its final result.

Otherwise the algorithm records the candidate best fit as the new current best fit and returns to Step 3 to search for a further improvement with additional parameters.

Upper bound on species-richness estimates

Any reconstruction of missing species using a small sample from a large population suffers from a fundamental limitation. Species that are too rare to have an appreciable chance of appearing in the sample cannot be estimated based upon the sample. As shown by Mao and Lindsay, this results in upper confidence intervals for the missing species that are formally infinite.

As discussed, Recon addresses this problem by only estimating those species that are large enough to have an appreciable chance of influencing the sample distribution in a meaningful way. (Note that while mixing distributions are often approximated as continuous, in reality they are discrete, so smallest fitted population will often be practically meaningful.) In many cases this estimate will be of interest.

But this still leaves the estimate for *all* species unbounded. The number of individuals in a population is of course an upper bound for the number of species. In many cases of interest, such as analysis of immune repertoires, it is relatively easy to obtain reasonable estimates of the total number of individuals. For example, an estimate of total cells can be obtained by scaling a cell count against total tissue or blood volume, e.g., 10^{10} B cells in the body.

Below we show how the Recon fit can be combined with an estimate of the number of all individuals in a population to get a sharper upper bound on the number of all species.

Recon produces an overall clone-size distribution. The smallest clone size in this distribution is described by two parameters: the fraction of all clones that are of this size, w_{min}, and a mean number of cells that it contributes to the sample, m_{min}. Clone sizes smaller than this contribute a mean of zero cells to the sample; however, it is possible that there are smaller clones in the parent population, clones so small that they both do not contribute to the sample and are invisible to our algorithm. Recon's estimate of the number of missing clones would not count such clones because it is not necessary to assume that they exist in order to obtain the observed sample clone-size distribution. However, if they were to exist, they would result in an undercount

of the species richness in the parent. The goal in this section is to bound this potential undercount. One can then test its plausibility, as described in the main text.

The maximum undercount U_{max}, and therefore the desired upper bound, is obtained for the case that all the cells in clones smaller than m_{min} are actually singlets. How many would that be? The answer is given by

$$U_{max} = R w_{min} m_{min} N/S$$

where R is (Recon's upper bound of) the overall species richness estimate, N is the total number of cells in the overall repertoire, and S is the sample size. Note the ratio S/N is the fraction of cells in the overall population that are sampled; scaling m_{min} by S/N (yielding $m_{min}*N/S$) thus gives the smallest clone size in the overall repertoire that Recon can distinguish from singlets. Error bars on R and uncertainty in N contribute to uncertainty in the upper bound. Because generally $N > R$, upper bounds are larger than Recon's estimates. We note, however, that in our experimental datasets (see main text) comparison of upper-bound estimates to the error expected given the coverage (S/U_{max}) excludes U_{max} as a plausible estimate, given the observed R (Fig. 4d).

An example of a limiting case will illustrate how the formula for U_{max} works. Suppose an organism contains $N = 10^{10}$ B cells, and further suppose that every one of these is a distinct clone, so that each clone in the parent is made up of 1 cell. If a sample of $S = 10^6$ cells is taken, then the observed clone size distribution will consist of 10^6 singletons, i.e. n_1 = 1,000,000 and remaining $n_i = 0$. The best that Recon could do here would be to take a single population (that is w =1.0) and note that the mean contribution, m, of each clone in the overall repertoire must be less than 10^{-3}.

The value of m comes from the fact that no clone is observed twice, so that $(10^{-3})^2 * S < 1$. Note that in fact the true mean contribution of each clone to the sample is 10^{-4}. Taking $m = 10^{-3}$ will result in a severe undercount, but is all that can be said with confidence given the sample size.

The unseen species estimated by Recon will be given by

$$n_0 = \frac{n_{obs}}{1 - p_0}.$$

In this example $n_{obs} = S = 10^6$. Recon's estimate of p_0 will be given by $1-p_{>0}$, where $p_{>0}$ is the chance that a clone contributes to the sample. Therefore the estimate of $1- p_0$ will be $p_{>0} = 10^{-3}$. Again, the true value of p_0 is much greater, but this is the best estimate possible given the sample. This results in an estimate

$$n_0 = \frac{10^6}{10^{-3}} = 10^9$$

The estimate of the species richness R is then $S + n_0$, which is approximately 10^9. In this extreme case, Recon therefore underestimates the true species richness by a factor of 10.

However, the formula for U_{max} is able to recover the true population. Since Recon fits only a single weight and mean, $w_{min} = w = 1.0$ and $m_{min} = m = 10^{-3}$. Then

$$U_{max} = \frac{10^9 \times 1.0 \times 10^{-3} \times 10^{10}}{10^6} = 10^{10}.$$

As expected, in this case Recon adds no further constraint. If every individual in the sample is from a different species then the only sensible upper bound for the number of species is N.

Now consider a case in which $S = 10^6$ cells are again sampled, but now the observed distribution has $n_1 = 900{,}000$, $n_2 = 35{,}640$, $n_3 = 6{,}667$, $n_4 = 1{,}500$, $n_5 = 400$, $n_6 = 100$, $n_7 = 10$, $n_8 = 5$, $n_9 = 1$ and remaining $n_i = 0$..

In this case the sample contains 944,323 clones. Recon fits 19,919,406 missing species for a total richness of 20,863,729 detectable clones. The w_{min} of the fit is 0.252 and the m_{min} is 0.041. The upper bound is now:

$$U_{max} = \frac{2.09 \times 10^7 \times 0.252 \times 0.041 \times 10^{10}}{10^6} = 2.16 \times 10^9.$$

The upper bound of N can therefore be usefully reduced by a factor of almost 5.

Power calculations for species richness

To obtain the minimum number of cells suggested to power an experiment detecting a specified difference, we required a number of cells sufficient to separate the expected sample means by at least one error bar, where the error bar is calculated as described in the main text.

If experimentally reconstructed missing species from multiple identical samples with identical true overall diversity are taken to be normally distributed, then our calculation corresponds to a t-test at p=0.05 using our error bar as an estimate of the 3 times the standard deviation of this distribution.

Further comparisons and non-identifiability

It has been observed that very similar models (reconstructions) of overall populations may nevertheless yield very different estimates of overall species richness[1]. To illustrate the point, Link[1] fit seven different models (A-G) to a data set for which the true overall species richness, N, is

unknown, with results shown in the following table (Table 3 of Ref. 1; ranges correspond to 95% profile likelihood [PLI] intervals for A-G and error bars for Recon):

model	Estimate of *N*	Est. error	χ^2	*df*
A	2,571 (2,554-2,589)	0.143	250,000	13
B	2,776 (2,730-2,827)	0.075	206	11
C	2,930 (2,840-3,025)	0.023	4.36	9
D	2,992 (2,867->10,000)	0.002	0.21	7
E	3,111 (3,018-3,218)	0.037	2.40	12
F*	3,320 (3,174-3,477)	0.107	0.94	12
G	3,494 (3,308-3,730)	0.165	2.71	12
CE	2,932 (2,855-3,026)	0.023	—	—
Recon	3,014 (2,709-3,513)	0.005	0.65	9

*Estimate based on correction of a typo in Ref. 1

Link notes that models A and B fit the data poorly, but the remaining models all fit well while yielding contradictory inferences about *N*. Notably, Model G fits this particular data set "extraordinary well," but on further investigation of synthetic data sets of this sort he finds that the lower 95% PLI of this model typically overestimates the true value of *N* [1].

Taken together, Link argues that these examples, particularly the fact that the 95% PLIs do not all overlap, confirm the "empirical observation" of Cormack that "many different forms can be found to fit the truncated distribution, while giving rise to vastly different estimates for the zero-frequency class."

We produced a Recon fit to Link's data set (in the table above; CE is calculated for comparison). The resulting estimate falls between the low and the high estimates of Link's well fitting models. The range reported by Recon is wider than the typical 95% profile likelihood estimates while providing useful information in excluding the bad fits A and B and the probable overestimate from model G. We conclude that the single Recon fit provides a reasonable summary of the estimates that would be made on investigation of these seven independent models.

To what extent do these examples show "substantially different inferences" or "vastly different estimates" of *N*? It could be argued that they show fairly similar estimates. However, this can question only be answered by reference to the practical purpose for which the estimate is made. For some applications Recon's error-bar range of 2,709 to 3,513 will be a "vast" or "substantial" difference. We argue that for applications in immune repertoire sequencing and many others ranges of this sort are not vast—they provide practical, useful information. Furthermore, the example brought forward by Link[1] illustrates that the error ranges reported by Recon effectively account for non-identifiability of *N* due to model mis-specification.

Supplementary References

1. Link, W.A. Nonidentifiability of population size from capture-recapture data with heterogeneous detection probabilities. *Biometrics* **59**, 1123-1130 (2003).
